# Hox Proteins in the Regulation of Muscle Development

**DOI:** 10.3389/fcell.2021.731996

**Published:** 2021-10-18

**Authors:** Gabriela Poliacikova, Corinne Maurel-Zaffran, Yacine Graba, Andrew J. Saurin

**Affiliations:** Aix-Marseille University, CNRS, IBDM, UMR 7288, Marseille, France

**Keywords:** Hox, muscle, patterning, mesoderm, neuromuscular

## Abstract

Hox genes encode evolutionary conserved transcription factors that specify the anterior–posterior axis in all bilaterians. Being well known for their role in patterning ectoderm-derivatives, such as CNS and spinal cord, Hox protein function is also crucial in mesodermal patterning. While well described in the case of the vertebrate skeleton, much less is known about Hox functions in the development of different muscle types. In contrast to vertebrates however, studies in the fruit fly, *Drosophila melanogaster*, have provided precious insights into the requirement of Hox at multiple stages of the myogenic process. Here, we provide a comprehensive overview of Hox protein function in *Drosophila* and vertebrate muscle development, with a focus on the molecular mechanisms underlying target gene regulation in this process. Emphasizing a tight ectoderm/mesoderm cross talk for proper locomotion, we discuss shared principles between CNS and muscle lineage specification and the emerging role of Hox in neuromuscular circuit establishment.

## Introduction

Homeotic or *Hox* genes are highly conserved homeodomain transcription factors that play a fundamental role in bilaterian animal body patterning (Pearson et al., 2005). Several characteristics are at the core of Hox gene function. First, Hox genes are differentially expressed along the anterior–posterior axis of the embryo allocating distinct morphological identities to each body part. Second, manipulation of their expression often results in spectacular homeotic transformations, where the morphology of one given body part is transformed into that of another. Third, Hox genes are grouped in clusters: one split cluster in *Drosophila*, the *Antennapedia* complex, ANT-C, harboring the Hox genes *lab*, *pb*, *Dfd*, *Scr*, *Antp* and the *Bithorax* complex, BX-C, harboring the Hox genes *Ubx*, *abd-A*, *Abd-B*; and at least four clusters in vertebrates (*HoxA-HoxD*) with each cluster harboring 1–13 Hox genes. Genomic clustering of Hox genes in such a way is essential for their correct spatio-temporal expression, which is controlled by important regulatory elements located within and around these clusters. Fourth, they all bind to a very similar set of “AT”-rich DNA-binding sites, achieving functional specificity by cooperating with transcriptional cofactors, the best characterized being PBC proteins (Extradenticle/Exd in *Drosophila*) and MEIS proteins (Homothorax/Hth in *Drosophila*), also encoding for homeodomain transcription factors. However, a large number of Hox-PBC/Meis independent functions have been reported and reciprocally, PBC/Meis proteins can function without binding to Hox (Mallo and Alonso, 2013; Rezsohazy et al., 2015).

Besides their canonical role in providing spatial coordinates that pattern the developing embryo, Hox genes play “non-homeotic” roles where they are involved in the regulation of virtually all basic cellular processes including cell death, cell proliferation, migration and differentiation, as well as in the development of whole structures/organs (Hombría and Lovegrove, 2003; Sánchez-Herrero, 2013). Yet relatively little is known into how much Hox proteins play a role in specification and development of vertebrate muscles, undoubtedly in part due to the large muscle diversification that exists in vertebrates, the large number of Hox genes present (more than 30), and a significant functional redundancy between paralogous group members. In *Drosophila melanogaster* however, there is now abundant evidence for Hox involvement in the patterning of several mesodermal derivatives, including somatic, cardiac and visceral muscles. It is now widely accepted that *Drosophila* myogenesis recapitulates, even though at different scales, all major muscle developmental events that are also at work in vertebrates, such as progenitor specification, myoblast migration and fusion, muscle attachment to tendons cells and assembly of contractile sarcomeres. Furthermore, a part of gene regulatory networks crucial for correct myogenesis such as *twist*, *mef2*, *lbx/ladybird* and the fusion machinery are well conserved (Schnorrer and Dickson, 2004; Taylor, 2006). Here, we focus specifically on Hox function in muscle precursor specification and in patterning of mesodermal derivatives, highlighting Hox target genes and their regulatory mechanisms, when available. We separate the topic in three sections, somatic/skeletal, cardiac and visceral muscles and in each section, we review available data from first *Drosophila* and then from vertebrates. In the somatic/skeletal section we further discuss an emerging role of Hox in the establishment of proper muscle-motoneuron connections. We distinguish Hox specific and non-specific, so-called generic functions, the first referring to functions performed by a single Hox that cannot be assumed by any other and the latter to functions that can be performed by several Hox genes (reviewed in Saurin et al., 2018).

## Somatic/Skeletal Musculature

### Basics on Somatic/Skeletal Muscle Development

The somatic muscle development in *Drosophila* as well as the functional conservation with vertebrate muscles have been extensively reviewed (Taylor, 2006; Dobi et al., 2015; Schulman et al., 2015; Gunage et al., 2017; Poovathumkadavil and Jagla, 2020). Briefly, the *Drosophila* life cycle comprises two mobile stages, the larval stage where the crawling movements enable larval feeding and the adult stage where flies can fly, jump and walk. Distinct sets of muscles, produced by two rounds of myogenesis are used during each stage, with larval muscles being produced during embryogenesis and the adult muscles during metamorphosis. Interestingly, both groups develop from mesoderm-derived somatic muscle progenitors marked by high *twist* expression, even though at different developmental timepoints (Baylies and Bate, 1996). Embryonic muscle progenitors are singled out from so-called equivalence groups or promuscular clusters in each hemisegment by lateral inhibition via Notch signaling at stages 11–12 (Carmena et al., 2002). The remaining cells of the cluster become fusion-competent myoblasts, providing mass to the growing muscle by fusing with it. After specification, muscle progenitors undergo a symmetrical division producing two muscle founder cells (FCs), or an asymmetrical division producing either one FC and one adult muscle progenitor (AMP) or one FC and one pericardial progenitor (Ruiz Gómez and Bate, 1997; Carmena et al., 1998). FCs seed individual embryonic muscles and express a unique combination of identity transcription factors (iTFs), controlling all muscle characteristics, including muscle size, position, innervation and attachment. During embryonic stages 12–15 fusion competent myoblasts fuse with FCs assuring muscle growth. Until larval hatching, muscles are properly oriented, attached to tendons and innervated.

**FIGURE 1:**
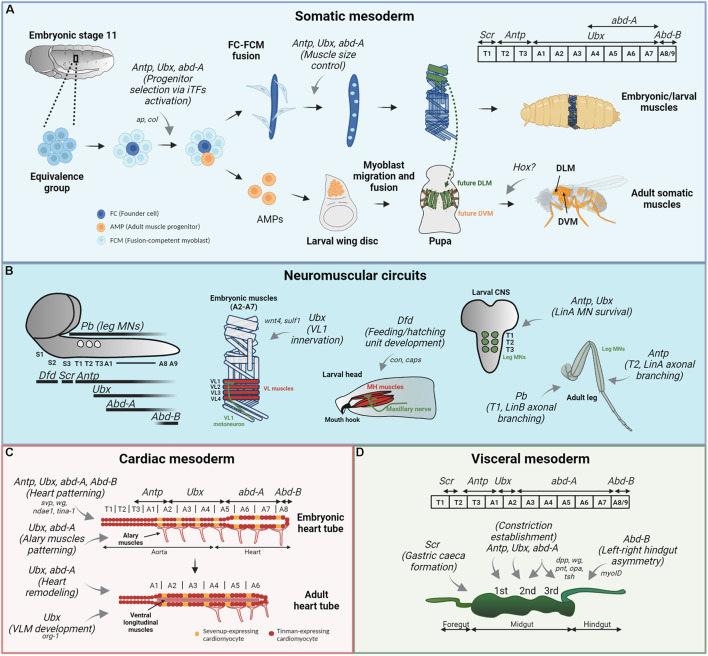
Hox functions in *Drosophila* muscle patterning. Hox functions in the somatic **(A)** mesodermal patterning, neuromuscular circuit establishment **(B)** and cardiac **(C)** and visceral **(D)** mesodermal patterning are depicted by gray arrows, and their target genes are specified, if available. Their expression patterns are showed for each mesodermal derivative. **(A)** In the somatic muscle development, Antp, Ubx, and AbdA regulate progenitor selection from different equivalence groups (by binding to a muscle specific *cis*-regulatory region of *ap* and *col*, in LT and DA3 muscles, respectively) and later control muscle size by specifying the number of FCM (light blue cells) that will fuse with the FC (dark blue cell). The role for Hox in adult musculature, derived from AMPs (orange cells) is not clearly established. **(B)** Hox expression pattern in the larval CNS is depicted on the left, with Pb expression in leg MNs (circles). In this context, Ubx establishes proper connection between embryonic VL1 muscle and VL1-MN, by regulating *wnt4* and *sulf1* expression in the VL2 muscle. Dfd is responsible for the feeding and hatching motor unit development and maintenance in the larva, a process involving target genes *con* and *caps*. Antp and Ubx play a role in LinA lineage MN survival during larval development, and Antp further regulates correct morphology of this neurons in the adult T2 leg. Finally, Pb controls branching of some LinB MNs in T1 adult leg. **(C)** The heart tube is composed of *Svp*-expressing (orange cells) and *Tin*-expressing (red cells) cardioblasts and surrounding pericardial cells (not represented). Antp, Ubx, and AbdA are all involved in the patterning of embryonic heart tube, by controlling the expression of *svp* in the segment-specific manner. Furthermore, AbdA with Svp regulate *wg* expression in cardioblasts expressing Svp. AbdB on the other hand is a suppressor of cardiogenesis. Ubx and AbdA pattern alary muscles present in the abdominal segments. For proper heart remodeling, Ubx repression and AbdA function modulation are required. Finally, Ubx is involved in adult ventral longitudinal muscle (VLM) development. **(D)** In the embryonic visceral mesoderm, Antp establishes the first, Ubx and AbdA the second and AbdA the third midgut constriction. A complex genetic network involving *dpp*, *wg*, *pnt*, *opa*, and *tsh* is involved in the middle constriction development. Scr is involved in the formation of gastric caeca in the anterior midgut (caeca are not represented) and AbdB is responsible for the correct left-right embryonic hindgut asymmetric morphology via *myoID* regulation.

On the other hand, AMPs/myoblasts that retain high *twist* expression do not differentiate directly after their specification but are set aside in a quiescent state and associated with imaginal disks and nerves. During larval stages, myoblasts proliferate extensively until the beginning of metamorphosis where they migrate, fuse and differentiate at an appropriate body position to constitute the adult body musculature. For the development of the most prominent adult muscles, the indirect flight muscles (IFMs) composed of dorsal longitudinal muscles (DLM) and dorsal ventral muscles (DVM), two different strategies are employed: The DLMs that span the thorax antero-posteriorly develop by the fusion of myoblasts with three larval scaffolds that escape the histolysis process during first hours of pupal development; the DVMs, spanning the thorax dorso-ventrally, develop by *de novo* myoblast fusion without any scaffold, like in the case of the larval musculature (Fernandes et al., 1991). The larval and adult myogenic process are thus intimately linked since muscle progenitors giving rise to both larval and adult muscles are specified during embryogenesis (even though they do not differentiate at the same time), the fusion process seems to involve the same molecular players and certain larval muscles are reused for the development of a group of adult muscles, like the DLM flight muscles (Figure 1A).

In vertebrates, like in *Drosophila*, muscles develop from mesoderm-derived muscle progenitors (reviewed in Endo, 2015; Musumeci et al., 2015; Chal and Pourquié, 2017). Briefly, skeletal muscles develop from the paraxial mesoderm, bilaterally flanking the neural tube. The paraxial mesoderm in the trunk region transiently and progressively subdivides into somites, which are themselves compartmentalized into the dorsal epithelial dermomyotome (giving rise to muscles, dermis, and brown fat) and ventral mesenchymal sclerotome (giving rise to the axial skeleton, cartilage, and tendons). A layer of muscle precursors/myocytes called myotome forms beneath the dermomyotome at E8 in the mouse and E2.5 in the chick. Muscle precursors after their differentiation give rise to the body wall muscles and some undergo long-range migration toward future limb buds where they proliferate and differentiate into limb muscles. In the cranial region, paraxial mesoderm forms the cranial mesoderm that will give rise to the muscles, connective tissue and skeleton of the face and skull. The myogenic process can be subdivided into primary/embryonic myogenesis (E10.5–E12.5 in mice and E3–E7 in the chick) and secondary/fetal myogenesis (E14.5–E17.5 in mice and E8+ in the chick). In vertebrates, individual muscles are not seeded by a single founder myoblast like in *Drosophila*, but by scaffolds of primary fibers composed of several fused muscle precursors. Subsequently, additional fibers are added along them to assure the muscle growth.

### Hox Control of *Drosophila* Embryonic/Larval Somatic Musculature

In the embryonic somatic mesoderm, *Hox* gene expression has a spatially restricted pattern, where *Sex combs reduced* (*Scr*) is expressed in the first thoracic segment, T1, *Antennapedia* (*Antp*) in T2 and T3, *Ultrabithorax* (*Ubx*) in abdominal segments A1–A7, *abdominal-*A (*abd-A*) in A4–A8 and *Abdominal-B* (*AbdB*) in A8–A9 (Michelson, 1994; Enriquez et al., 2010; Figure 1A). Their implication in the subdivision of somatic mesoderm began with the description of mutant alleles of *AbdB* (Lawrence and Johnston, 1984) and *Ubx* (Hooper, 1986) shown to transform larval muscles into more anterior ones. Conversely, the overexpression of Ubx or AbdA in the embryonic mesoderm led to the abdominal transformation of thoracic muscle precursors (Greig and Akam, 1993; Michelson, 1994). Collectively, these studies demonstrated a clear role for Hox genes in muscle patterning and argued toward a cell-autonomous role of Hox in the somatic mesoderm, meaning that Hox genes would confer a specific identity to the muscle precursors and account for segment-specific differences in the muscle pattern. Further evidence for cell-autonomous Hox function came from a pioneering study showing that Antp directly regulates the expression of the *apterous* muscle identity gene in the somatic mesoderm, specifying a subset of lateral transverse muscles (LT1–4) (Figure 1A). Importantly, it was shown that this regulation occurs via an *apterous* muscle-specific enhancer, providing a molecular mechanism by which a Hox protein can directly regulate its muscle target gene expression and contribute to the establishment of segment-specific muscle patterns via progenitor selection (Capovilla et al., 2001). Whether Ubx/AbdA specify abdominal LT1–4 muscles and/or regulate *apterous* expression was not addressed.

Building on the same principle, it was found that Antp, Ubx, and AbdA regulate progenitor selection via distinct *cis*-regulatory modules in a segment-specific manner (Figure 1A). Focusing on the dorsal acute DA3 muscle lineage, specified by the combinatorial activity of *nautilus* (*nau*, *Drosophila* ortholog of mammalian *MyoD*) and *collier* (*col*, ortholog of mammalian early B-cell factors, EBFs) it was shown that the progenitor specification via Hox-mediated regulation of muscle iTFs is superimposed on the A-P and D-V positional information. Furthermore, the precise timing of Hox activity was traced to the progenitor stage, where first, Hox activity controls the number of progenitors that express *col* and *nau* in a segment-specific manner and then Hox proteins interplay with iTFs to allocate a correct number of nuclei assigned to the DA3 muscle (Enriquez and Vincent, 2010; Enriquez et al., 2010; Figure 1A). Interestingly, this study highlighted a Hox generic function, where Antp, Ubx, and AbdA can all provide DA3 progenitor selection (reviewed in Saurin et al., 2018). This generic function is then followed by a Hox specific function, where each Hox determines the final size of the muscle in a segment-specific manner. These studies provided an important insight into the function of Hox genes in myogenesis, showing that Hox inputs were crucial in the process of progenitor selection, which gives rise to “founder” cells, seeding the formation of syncytial muscle fibers.

It was recently shown that Ubx is involved in the muscle differentiation process by directly repressing the master mesodermal regulator *twist* (*twi*) (Domsch et al., 2021). Here, a number of interesting points can be highlighted: (1) Ubx mesodermal downregulation interferes with abdominal embryonic muscle development because of failure to repress *twi*; (2) *twi* repression is mediated by direct Ubx binding to the *twi* promoter region, competing with binding of the transcription factors Tinman (Tin) and Muscle enhancer factor 2 (Mef2), providing another promoter-based molecular evidence about the Hox muscle patterning mechanism; (3) even though Ubx binding displaces Tin on the *twi* promoter, Tin must be bound to the promoter for Ubx to be recruited, once again demonstrating a tight link between Hox and tissue-specific transcription factors for the correct patterning establishment.

Collectively, these studies provide precious insight into the way muscle progenitors are patterned. Hox proteins activate and collaborate with some of the muscle iTFs in a segment-specific fashion and by doing so they are responsible for the specification of different muscle types.

#### Is Patterning of the T2 Mesoderm Hox Independent?

As the understanding of muscle development evolved, an important role in muscle patterning was attributed to the ectoderm. A large number of studies argued toward a Hox non-autonomous role in myogenesis, giving importance to Hox-controlled signals coming from the overlying epidermis and nervous systems (Roy and Raghavan, 1997; Roy et al., 1997; Rivlin et al., 2001; Dutta et al., 2010). For a long time, the patterning of mesoderm in T2 was considered as being non-autonomous, because of the lack of any Hox gene expression in this segment. In complement to this observation, it was shown that overexpression of Antp (highly expressed in T2 and T3 epidermis and nervous system) in the ectoderm of *Antp* mutant embryos completely rescued the disorganized T2 muscle pattern provoked by Antp loss-of-function (Roy et al., 1997). This was taken as evidence for a non-autonomous role of Antp in T2 muscle pattern establishment. However, Antp was later described as being expressed in the T2 mesoderm, although at a much weaker level than in the T3 mesoderm and to autonomously specify a subset of muscles in the T2 (Capovilla et al., 2001). Thus, the default in T2 muscle patterning in *Antp* mutant embryos is either pointing toward an autonomous role of Antp in the mesoderm or toward a combination of autonomous activity and inductive cues coming from the T2 ectoderm. It was also shown that Antp mesodermal overexpression did not modify T3 muscle pattern, but was sufficient to transform the T2 into a T3 muscle pattern, which was interpreted as another evidence for the lack of Antp T2 expression (Roy et al., 1997). Knowing that Antp expression in the T2 mesoderm is weaker compared to that of T3, higher levels of Antp in T2 may convert its muscle pattern into a more posterior one, without excluding the presence of Antp in T2 mesoderm.

### Hox Control of *Drosophila* Adult Musculature

As mentioned earlier, *Drosophila* adult musculature develops from pools of myoblasts associated with imaginal tissues and nerves. The most prominent adult muscles, thoracic flight muscles arise from myoblasts associated with the wing imaginal disks. Knowing that T2 mesoderm was mistakenly considered as a Hox-free region because of lack of *Antp* expression, it is possible that T2 wing disk-associated myoblasts also express Antp. In support of this, it was reported that Antp is transcribed from two distinct promoters, termed P1 and P2, with transcripts from P1 being localized to the anterior part of the wing disk epithelium and P2 transcripts in the presumptive notum region containing myoblasts (Jorgensen and Garber, 1987). Recently, antibody stainings have confirmed the presence of Antp protein in the myoblast-containing region of the wing disk (Paul et al., 2021). If indeed the expression of Antp is confirmed by appropriate markers in adult muscle precursors [such as Mef2, Holes in muscles (Him), or Twi], then it is highly possible that Antp is directly involved in T2 adult muscle development, including muscles used for flight and leg muscles.

Early myoblast transplantation experiments showed that T2 myoblasts, associated with the wing imaginal disks, can contribute to a vast population of adult muscles (Lawrence and Brower, 1982). Since T2 wing-associated myoblasts were considered as a Hox-free region (see above), it was proposed that they do not require any positional Hox input for their migration and fusion. Supporting this view, mesodermal overexpression of Ubx does not perturb thoracic myoblast migration pattern, demonstrating that the myoblast migration process is likely independent of Hox positional input. These myoblasts however fail to give rise to a proper adult flight muscle, because of their inability to activate Act88F, a fibrillar muscle differentiation marker, that is repressed due to Ubx overexpression (Roy and Raghavan, 1997).

Concerning myoblast fusion, the importance of Hox cues has not yet been clearly established. It is important to note that myoblasts transplanted from the second or the third thoracic segment, associated with wing and haltere imaginal disks, respectively, can both fuse to and contribute to abdominal muscles (Roy and Raghavan, 1997). It is thus possible that Antp could be involved in the thoracic myoblast fusion process, a hypothesis that has never been directly addressed because of a lack of appropriate tools. Rivlin et al. (2001) demonstrated using allelic combinations of Ubx mutations, leading to different levels of ectodermal transformation, that the transformed T3 segment could contain IFM, normally present solely in T2 (Rivlin et al., 2001). This placed ectodermal inductive cues in the central position of adult IFM patterning. However, this observation has not been reproduced (Egger et al., 1990; Fernandes et al., 1994; Dutta et al., 2010), leaving the role for Hox proteins in adult muscle development not elucidated. We note that no Hox regulatory networks were identified for the development of adult abdominal and leg muscles, leaving the role of Hox in adult muscle development largely undetermined.

It is noteworthy that the well-characterized Hox PBC cofactors, Exd and Hth are involved in the adult muscle development, controlling the fate decision between fibrillar flight and tubular leg muscles, but appear to do so in a Hox-independent fashion (Bryantsev et al., 2012). Knowing that for the proper somatic musculature patterning, Hox proteins use other muscle-specific transcription factors as cofactors, it is thus likely that Hox control of somatic muscle development is independent of their canonical cofactors and reciprocally, Exd/Hth function is independent of Hox proteins in this context.

### Hox Control of Vertebrate Skeletal Musculature

In vertebrates, Hox involvement in skeletal, myotome-derived musculature patterning remains controversial. In the limb musculoskeletal system, Hox genes have an essential role in skeletal and connective tissue patterning, but limb muscle precursors seem to depend completely on environmental cues for their proper development (reviewed in Pineault and Wellik, 2014). Early grafting experiments in the chick/quail embryos demonstrated that muscle precursors do not possess intrinsic patterning information and their development is influenced by the surrounding mesenchyme (Chevallier et al., 1977; Christ et al., 1977). This view has been later challenged by a study suggesting that the axial identity of the somite is important for the generation of non-limb skeletal muscles, arguing toward an autonomous role for Hox in the body wall muscle patterning (Nowicki and Burke, 2000; Alvares et al., 2003). Furthermore, microarray analyses on purified skeletal muscle myoblasts showed that the Hox code is present along the cranio-caudal axis, specifically in embryonic, but not fetal myoblasts, indicating that myoblasts carry some intrinsic positional information (Biressi et al., 2007).

Direct evidence for Hox role in skeletal muscle patterning came from the mouse forelimb zeugopod, where Hoxa11 and Hoxd11 are expressed in the muscle connective tissue and tendons, but not in differentiated muscle cells (Swinehart et al., 2013). In Hoxa11/d11 double mutant mice, several muscles and tendons of the forelimb zeugopod are absent or improperly patterned, and importantly, this is a direct consequence of loss of Hox function and not a secondary effect due to defects in the skeletal patterning. In addition to extending the well-established role of Hox in the patterning of the axial skeleton to muscles and tendons, this study also reinforces the initial view that muscle precursors are patterned by their environment, at least at the level of the limb. However, it is still unknown whether the Hox positional information is conveyed by the muscle mesenchyme or is encoded in muscle precursors themselves, which could be dependent on the somite axial level.

Recently, much attention has been given to the role of Hox proteins in adult muscle stem cells (MuSCs), also known as satellite cells, able to regenerate adult muscles upon injury (Machado et al., 2017; Evano et al., 2020; Yoshioka et al., 2021). It is now accepted that depending on the anatomical position of the muscle, satellite cells display heterogeneity in their proliferative and regenerative properties (Ono et al., 2010). Searching for underlying molecular determinants, Hox-A and Hox-C clusters were found to have different methylation profiles and thus be differentially expressed in adult muscles and their satellite cells derived from somites, compared to the ones derived from the cranial mesoderm (Evano et al., 2020; Yoshioka et al., 2021). Knowing that the cranial-derived, Hox-free, satellite cells display higher regenerative capacity than the limb, Hox-expressing satellite cells, an appealing possibility is that Hox could be involved in this process (Evano et al., 2020). Indeed, it has been demonstrated that during the aging process, *Hoxa9* is up-regulated in the limb satellite cells, leading to their cell cycle entry default and senescence (Schwörer et al., 2016). It does so by regulating the targets of several developmental pathways, including those of the Wnt, TGFβ, and JAK/STAT pathways. Interestingly, *Hoxa9* deletion in satellite cells from aged adult mice was sufficient to improve their regenerative capacity, suggesting that Hox expression in these cells would have a negative effect on muscle regeneration. A conditional depletion of *Hoxa10* leads to a repair default of some somite, but not cranio-derived muscles, explained by a genomic instability and consequent proliferation arrest of adult satellite cells (Yoshioka et al., 2021). Different Hox would thus assume opposed functions in satellite cell proliferation and could account for different capacities of distinct muscle groups to regenerate, a hypothesis that needs to be investigated further. In *Drosophila*, a muscle satellite cell population has been identified in adult thoracic flight muscles (Chaturvedi et al., 2017), but it remains unknown whether Hox proteins are involved in their transcriptional regulation like in vertebrates.

### Hox Control of *Drosophila* Neuromuscular Circuits

Hox involvement in central nervous system development is clearly established and is out of the scope of this review (Rogulja-Ortmann and Technau, 2008; Estacio-Gómez and Díaz-Benjumea, 2014; Meng and Heckscher, 2021). It is nevertheless noteworthy here that several parallels can be drawn between neuronal and somatic muscle lineage specification by Hox. In both tissues Hox act at the very early steps of progenitor specification. Like in the larval DA3 muscle where Hox allocate a correct number of progenitors and further control muscle size in a segment-specific manner, in the neuroectoderm their expression is required to specify NB1-1 derived thoracic and abdominal lineage comprising a different number of neurons (Prokop and Technau, 1994; Prokop et al., 1998; Enriquez et al., 2010). Hox can convey the proper tissue pattern via the regulation of specific TFs, shared across distinct tissues. For example, Antp regulates the expression of *collier* (*col*) in both muscle and neural clusters to generate muscle/neuronal diversity (Enriquez et al., 2010; Karlsson et al., 2010). Yet the transcriptional control mechanisms at play would appear different, since the *cis*-regulatory element used by Antp in the muscle lineage is distinct from the CNS regulatory sequence, which has to date not been identified. One important difference though, is that in the CNS, the Hox cofactors Exd and Hth are directly involved in the control of *col* expression. Like in the somatic and cardiac mesoderm, AbdB is able to suppress neuronal fate in the most posterior abdominal segments (Lovato et al., 2002; Ponzielli et al., 2002; Birkholz et al., 2013).

Crosstalk between neuronal and muscle lineages, more specifically between motor neurons (MNs) and somatic/skeletal muscles are at the basis of voluntary movements, crucial for locomotion, feeding, mating and interactions with the environment. For their establishment, MNs need to be correctly specified and differentiated and project their axons toward specific muscle groups. Neuromuscular circuitry defaults are associated with numerous neuromuscular diseases, thus the understanding of their proper development has a direct clinical significance (reviewed in Tripodi and Arber, 2012; Li et al., 2018).

In the CNS, Hox expression is shifted posteriorly compared to their mesodermal expression. Dfd is expressed in two subesophageal segments (S1 and S2), Scr is expressed in S3, Antp is expressed in the ventral cord in T1 to A9, Ubx from T3 to A9, Abd-A from A1 to A9 and Abd-B from A7 to A9. Pb is expressed in the thoracic leg motoneurons (Baek, 2011; Figure 1B). Hox involvement in neuromuscular circuitry establishment was first proposed in the context of larval crawling movements, following the observation that Ubx and AbdA are necessary for their generation, providing a genetic explanation for locally specialized locomotor circuit establishment (Dixit et al., 2008). This study suggested that Ubx and AbdA not only specify larval abdominal muscles required for these peristaltic movements, but also the neuronal circuitry allowing for the properly synchronized movements. Moreover, the single removal of either Ubx or AbdA did not compromise the peristaltic movement, demonstrating a genetic redundancy in Hox function since Ubx and AbdA can substitute for each other in this context. Ubx and Antp have been shown to be required for motoneuron segmental diversity in the embryo (lineages NB7-3 and NB2-4t), by regulating cell-death and cell-survival, respectively, in an antagonistic manner (Rogulja-Ortmann et al., 2008). Several Ubx and Antp putative binding sites were identified in the pro-apoptotic *reaper* (*rpr*) gene enhancer, suggesting that their competitive binding could account for their opposed regulatory mode of motoneuron survival.

An example of a molecular mechanism behind the role of Ubx/AbdA in locomotor circuit establishment has been elucidated in embryonic abdominal ventrolateral (VL) muscles. Ubx is expressed in both muscle cells and MNs and is required for the correct establishment of contacts between them (Figure 1B). In the VL2 muscle, it controls the activation of Wnt4 signaling as it does in the visceral mesoderm (Graba et al., 1995), and the expression of Sulf1, a sulfatase implicated in Wnt and BMP gradient establishment at neuromuscular junctions. In the VL1 MN, Ubx interacts with the components of the Wnt4 pathway. Signaling molecules regulated by Ubx in VL2 upon their secretion serve for the proper VL1 MN axonal extension toward the more dorsal VL1 muscle. Interestingly, it was suggested that Ubx, TCF, and Armadillo (Arm) can form a Wnt4-induced transcriptional complex (Hessinger et al., 2017). The requirement of Hox in both muscles and MNs was also demonstrated in larval hatching and feeding motor units, with the central Hox, *Dfd* (Friedrich et al., 2016). Here, Dfd assures correct innervation of muscles required for mouth hook movements by the maxillary nerve (Figure 1B). This demonstrates that Hox could provide a regulatory code for the correct muscle-motoneuron recognition in different *Drosophila* neuromuscular circuits. In line with this hypothesis, taking advantage of available ChIP-seq data, several potential Dfd target genes with functions in muscles, nerves and synaptic recognition were identified, such as *Connectin* (*Con*) and *capricious* (*caps*) (Friedrich et al., 2016).

Concerning adult muscle innervation, the role of Hox is well established in the case of leg muscles, where the second leg pair is required for flight take-off, and T1 and T3 legs are required for grooming the head and the abdomen, respectively (Baek et al., 2013; Enriquez et al., 2015). Focusing on a major subpopulation of leg MNs, arising from the LinA (also called Lin15B) lineage, it was shown that during the third larval stage, Antp is expressed in the newborn LinA MNs in all three thoracic segments, whereas Ubx was localized only in T3 subpopulation. During mid-pupal stage, the expression of Hox changes, with Antp expression being confined to all LinA MNs solely in T2 and Ubx solely in T3 and this segment-specific expression pattern is maintained until the adult stage. Hox cofactors Exd and Hth are ubiquitously present from the larval until the adult stage. In this context, Antp and Ubx with their cofactors are required for LinA MN survival, and Antp with Hth are further required for the correct axonal and dendritic morphology and axonal branching (Figure 1B). We note that Hox proteins do not specify leg motoneurons *per se*, but assure instead motoneuron survival and offer a unique code for their correct branching to distinct leg parts. Interestingly, this highlighted the importance of Antp protein levels, serving as a timing mechanism for correct proximal (early born) vs. distal (late-born) axon targeting (Baek et al., 2013). Antp protein levels seem to play an instructive role as well in T2 vs. T3 somatic muscle pattern establishment (as mentioned above), suggesting that several Hox functions could be dose-dependent.

While looking for a genetic explanation for distinct morphological characteristics of individual motoneurons, an important role was attributed to anterior Hox *proboscipedia* (*pb*). Concentrating on the LinB (Lin24) lineage of leg MNs, it was demonstrated that Pb, expressed in three of seven neurons composing this population, is required for their proper dendritic morphology and axonal patterning (Figure 1B). Clonal removal of *pb* in T2 LinB MNs affected the linearity of path/stability during high speed walking (Enriquez et al., 2015).

A role for Hox in adult muscle innervation was also shown with Ubx whose misexpression in T2 MNs compromised adult IFM development (Dutta et al., 2010). Collectively, muscle development and homeostasis require both Hox autonomous and non-autonomous function, in the *Drosophila* mesoderm and neuroectoderm, respectively.

### Hox Control of Vertebrate Neuromuscular Circuits

In vertebrates, abundant evidence argues toward an important Hox function in motor circuit establishment (reviewed in Jung and Dasen, 2015). For the correct locomotor circuitry establishment, a large variety of different MN subtypes need to form precise connections with target muscles. A well-studied group of MNs, spinal MNs display different columnar, divisional and pool identities allowing them to contact more than 50 different limb muscles. Concentrating on chick brachial lateral motor columnar (LMC) neurons, it was shown that Hox3, 4, 5, 7, and 8 proteins divide LMC into subdomains along the rostrocaudal axis. Importantly, changing MN transcriptional identity by manipulation of Hox protein levels resulted in corresponding changes in muscle connectivity. It has been proposed that in this context, Hox proteins confer different pool identities by regulating downstream transcription factors in MNs, such as Nkx6 (NK6 Homeobox 1) (Dasen et al., 2003, 2005). Hox6 proteins do not have a role in the initial LMC specification but are required for further LMC pool identity establishment and proper limb innervation (Lacombe et al., 2013). In the search for the link between neuronal identity specification by Hox and muscle innervation in chick and mice, the transcription factor FoxP1 (Forkhead box protein P1) was identified as a Hox accessory factor, allowing to fine-tune Hox output (Dasen et al., 2008). The molecular mechanism behind this process also involves cell surface receptor encoding genes *Ret proto-oncogene* (*Ret*) and *Glial cell line-derived neurotrophic factor receptor alpha* (*Gfr*α). In this context, Hox proteins in collaboration with their cofactor Meis1 were shown to regulate the spatial pattern and expression levels of these genes in LMC neurons, required for proper MN differentiation and connectivity (Catela et al., 2016). Interestingly, digit-innervating MNs in chick and mice also employ a Hox code for their specification, that is however different from the one used in more proximal limb muscles. In this context, joint expression of Hoxc8 and Hoxc9 are required for correct digit innervation (Mendelsohn et al., 2017).

Hox proteins not only control LMC neuromuscular circuitry at the limb level, but also at the thoracic level, where MNs innervating hypaxial muscles are specified by Hoxc9, acting as a repressor of a limb-innervating MN fate (Jung et al., 2010). Non-limb innervating MNs at the cervical spinal cord level within the phrenic motor column (PMC) also require Hox for their correct development. Interestingly, mice lacking *Hox5* genes (*Hoxa5* and *Hoxc5*) in these neurons die of respiratory failure as a consequence of altered diaphragm innervation (Philippidou et al., 2012; Philippidou and Dasen, 2013). Furthermore, spinocerebellar tract neurons (SCTNs) that relay sensory/proprioceptive information to the CNS from muscles and tendons also use Hox-dependent transcriptional program for their diversification. Discrete populations of SCTNs along rostro-caudal axis display a combinatorial expression of several Hox genes (from *Hox4* to *Hox10*) and their genetic manipulation leads to defaults in muscle-cerebellum connectivity (Baek et al., 2019). Collectively, both spinal MNs and sensory SCTNs use Hox-code for their proper specification, suggesting a general role for Hox proteins in the proper muscle-neuron connectivity establishment.

## Cardiac Muscle

### Hox Control of *Drosophila* Cardiac and Alary Muscles

*Drosophila* cardiac musculature develops during embryogenesis from lateral mesodermal cells that migrate toward the dorsal midline during dorsal closure and form a tube named the cardiac tube, also known as the dorsal vessel (reviewed in Monier et al., 2007; Bataillé et al., 2015; Rotstein and Paululat, 2016). The cardiac tube is subdivided into two parts, with the anterior narrow portion (T1–A4) named aorta and the posterior larger one (A5–A8) named heart, with the hemolymph flowing from the posterior to anterior, assuring its distribution throughout the organism (Figure 1C). Aorta can be further subdivided into anterior (T1–T3) and posterior (A1–A4) parts. In the anterior/thoracic aorta, each segment contains four pairs of cardiomyoblasts (CMs) that express the homeodomain-containing transcription factor Tinman (*tin*) (Nkx2.5 in vertebrates). The posterior/abdominal part is constituted of six pairs of CMs, with two anterior ones expressing the orphan nuclear receptor Seven-up (*svp*) (ortholog of Nuclear Receptor Subfamily 2 members) and the four posterior CM pairs expressing *tin* (except A8 that contains only two *tin*-expressing CM pairs). *Svp*-expressing cardioblasts in the heart further differentiate into ostiae, inflow valves that allow for the hemolymph pumping, accounting for the partially open circulatory system in *Drosophila* (Molina and Cripps, 2001). The metamerically repeated expression of *svp*, *tin* and other genes suggested the cell-identity specification in a segment-specific fashion. During metamorphosis, the heart part of the cardiac tube is histolyzed, and the adult heart develops from the larval posterior aorta myocytes that undergo a transdifferentiation without cell proliferation (Monier et al., 2005).

*Hox* genes have a rather complex expression pattern in the dorsal vessel. The anterior aorta is considered as a Hox-free region. The posterior aorta expresses *Antp* (in part of the T3, in A1 and weakly in A2 CM) and *Ubx* (from A2 to A5 CM) and the heart expresses *abd-A* (from the fifth pair of A5 CM to the second pair in A8) and *AbdB* (in the two posterior CM pairs in A8) in both cardioblasts and at least some pericardial cells (Lo et al., 2002; Lovato et al., 2002; Ponzielli et al., 2002; Zmojdzian et al., 2018; Figure 1C). Hox were shown to be responsible for dorsal vessel patterning in a cell-autonomous fashion (reviewed in Monier et al., 2007). Independent studies demonstrated that Ubx and AbdA are responsible for heart cardioblast specification (Figure 1C). In *abd-A* null embryos the heart is transformed into the aorta and conversely, its mesodermal overexpression is sufficient to specify aorta as heart cardioblasts instead (Lo et al., 2002; Ponzielli et al., 2002). In *Ubx* or *Ubx/abd-A* double mutants, the anterior part of the aorta is affected (Ponzielli et al., 2002). Antp does not specify cardiac lineage *per se* but generates CM diversity by controlling *svp* expression in the A1 segment (Perrin et al., 2004). Interestingly, *AbdB* mesodermal overexpression suppresses cardiac fate while its loss-of-function leads to supernumerary cardioblasts but also somatic nuclei (Lovato et al., 2002; Ponzielli et al., 2002). The combined mutations of *Scr*, *Antp*, *Ubx*, *abd-A*, and *AbdB* transformed the whole dorsal vessel into the aorta, further showing that the aorta fate is a ground state of the cardiac tube (Perrin et al., 2004).

Even though Hox proteins clearly regulate the cell lineage choice between the anterior aorta and posterior aorta/heart only a few of their target genes involved in this process have so far been identified. Antp, Ubx, and AbdA control *svp* expression in their respective segments in the embryonic heart, a function that can be once again considered as generic (Perrin et al., 2004; Ryan et al., 2005). *Svp* itself was shown to be regulated by Hedgehog (Hh) signaling coming from the ectoderm. Interestingly, it was suggested that cardioblasts can respond to Hh signals only in Hox-expressing cells, explaining why the anterior aorta (a Hox-free region) does not express *svp* even though it receives Hh (Ryan et al., 2005). To explain heart-patterning control by Hox, it was suggested that Hox could regulate symmetric/asymmetric division of progenitors giving rise to *tin* and *svp*-expressing cardioblasts and pericardiac cells, although this hypothesis remains to be tested. AbdA regulates the expression of *Troponin C-akin-1* (*Tina-1*), a heart-specific marker, of unspecified function (Lovato et al., 2002). Some target genes were identified at later stages, where Hox further pattern the individual cardiomyoblasts. AbdA in collaboration with Svp was suggested to activate *Wg* expression in heart cardioblasts expressing *svp*. In the Tin-expressing cardioblasts, AbdA activates expression of a Na^+^-driven anion exchanger (*ndae1*), involved in ionic homeostasis (Perrin et al., 2004). No gene level mechanisms explaining the Hox cardiac target gene regulation have however been identified, leaving the possibility of the existence of tissue-specific *cis*-regulatory modules used by Hox, as is the case of the somatic musculature.

Interestingly, ecdysone-dependent repression of Ubx in A1-A4 *tin*-expressing myocytes is required during the mid-pupal stage for adult heart formation (Figure 1C). Adult heart develops during metamorphosis by a remodeling of the larval posterior aorta. If Ubx expression is maintained during pupal stages in posterior aorta *tin*-expressing cells, they adopt A5 characteristics, resulting in the adult remodeling alteration (Monier et al., 2005). This argues in favor of a hypothesis stipulating that in the process of organogenesis, Hox input is necessary for the activation of downstream signaling networks but once these are activated, Hox presence is no longer needed and can be even detrimental for the rest of the development (Hombría and Lovegrove, 2003). The modification of AbdA is also required for heart metamorphosis, but occurring at the functional instead of expression level, yet also in an ecdysone-dependent manner (Figure 1C). Instead of conferring heart fate to the CMs like AbdA does during embryogenesis, here it regulates the reprogramming of A5 segment that becomes the terminal chamber in the adult. It is interesting to note that the switch in AbdA function occurs also at the transcriptional level, where early during development AbdA regulates positively *Ih* (a voltage-gated ion channel) expression but represses it in the pupa (Monier et al., 2005). We highlight that Hth is expressed only in the anterior aorta which does not express any Hox (Perrin et al., 2004), and thus Hox function in the *Drosophila* cardiac tube, like in the somatic muscle development (Bryantsev et al., 2012) is independent of Exd/Hth cofactors.

Besides cardiac muscle, Hox also control the patterning of seven pairs of alary muscles (AMs), specialized skeletal muscle connecting the cardiac tube at the level of Svp-expressing pericardial cells, with the lateral exoskeleton (LaBeau et al., 2009; Bataillé et al., 2015; Figure 1C). AMs were recently shown to maintain the dorsal vessel at the tracheal trunk position (Bataillé et al., 2020). During metamorphosis, four AM pairs remain, originating from larval posterior AMs (Lehmacher et al., 2012). Interestingly, three anterior AM pairs undergo a process of dedifferentiation and give rise to adult ventral longitudinal muscles (VLMs) of unknown function (Molina and Cripps, 2001; Schaub et al., 2015). Two Hox genes are expressed in AMs at the embryonic stage, *Ubx* in the three most anterior pairs and *abd-A* in the four posterior AM pairs. Consistent with the Ubx expression pattern, in *Ubx* mutant embryos, 2–3 anterior AM pairs do not form. The absence of AbdA does not compromise posterior AM formation, probably because Ubx and AbdA functions are redundant in this context. Conversely, Ubx or AbdA overexpression leads to supernumerary AM (LaBeau et al., 2009). Furthermore, modulation of Hox expression is required for correct AM transdifferentiation, since overexpression of AbdA in anterior AMs, leading to Ubx suppression, prevents VLM formation (Figure 1C). Like in the case of cardiac tube remodeling, ecdysone signaling is also required for AM transdifferentiation (Schaub et al., 2015). However, it is not known whether in this case ecdysone pathway modulates Hox activity like during adult heart development. Concerning Hox target genes, *optomor-blind-related-gene-1* (*org-1*, ortholog of vertebrate T-box Transcription factor Tbx1) was proposed as being directly or indirectly regulated by Ubx during adult VLM formation (Schaub et al., 2015). Knowing that *org-1* is also required for embryonic AM development, it is possible that Ubx (and also AbdA) could regulate *org-1* also during this stage (Boukhatmi et al., 2014).

### Hox Control of Vertebrate Cardiac Muscle

Many similarities can be found between *Drosophila* and vertebrate cardiac myogenesis even though at a first glance they seem very distinct. Both are developed from mesodermal precursors that converge toward the midline to give rise to a linear, contractile tube, that is further looped and developed into a multi-chambered organ in vertebrates (Zaffran and Frasch, 2002; Zaffran and Kelly, 2012; Lescroart and Zaffran, 2018). In birds and mammalians, cardiogenic precursors/first heart field progenitors (FHF) converging at the anterior midline express Mesp1 (Mesoderm Posterior bHLH Transcription Factor 1) and form a so-called “cardiac crescent.” The cardiac crescent develops into a transient heart tube with an inner endocardial and outer myocardial layer that will mainly contribute to the left ventricle. The heart tube elongates by the addition of second heart field (SHF) progenitors, located in the pharyngeal mesoderm (itself formed by cells of both splanchnic and paraxial mesoderm). SHF segregates along the AP axis into posterior SHF, contributing to the atrial myocardium at the venous pole and anterior SHF (also called AHF) that contribute to the outflow tract (OFT) (connecting the ventricles to the future aorta) and the right ventricle at the arterial pole. The heart is finally shaped by rightward looping and myocardium expansion leading to the formation of four integrated cardiac chambers, two ventricles and two atria. A specialized population of neural crest cells (NCC) contribute to the development of large arteries and outflow septum. A large number of inductive signaling molecules have been linked with vertebrate cardiac development, such as NK homeodomain proteins (e.g., Nkx2.5, *Drosophila tinman* ortholog), GATA (*Drosophila pannier* ortholog) and T-box families.

In contrast to skeletal muscles, a role for Hox in vertebrate cardiac muscle development is very well established (reviewed in Lescroart and Zaffran, 2018). It was recently shown that anterior Hox genes (*Hoxa1*, *Hoxa2*, *Hoxb1*, and *Hoxb2*) are expressed in a subpopulation of Mesp1-expressing cardiovascular progenitors that seem to be the last to emerge from the primitive streak. Interestingly, progenitors that do not express Hox seem to be unipotent in contrast with Hox-expressing progenitors that are bipotent, contributing either to cardiomyocytes and smooth muscles or cardiomyocytes and endothelial cells (Lescroart et al., 2014, 2018). Hox genes (*Hoxa1*, *Hoxa3*, and *Hoxb1*) are also expressed in SHF progenitors and their expression patterns subdivide this cell population in distinct domains: *Hoxa1* and *Hoxb1* are expressed in AHF with different anterior limits and *Hoxa3* is expressed in posterior SHF. While these progenitors contribute to both poles of the heart, *Hoxb1*-expressing progenitors are found only in the proximal OFT and atria and cells expressing *Hoxa1* and *Hoxa3* only in the distal OFT and some regions of the atria. SHF cells are thus pre-patterned before their addition to the developing heart (Bertrand et al., 2011). The same Hox genes are differentially expressed along the rostro-caudal axis in cardiac NCCs (Bertrand et al., 2011; Lescroart and Zaffran, 2018).

Concerning the role of Hox in cardiac development, it has been suggested (although not directly demonstrated) that *Hoxb1* could play a role in cardiac progenitor migration from the primitive streak (Lescroart and Zaffran, 2018). Mice mutant for *Hoxa1* develop heart patterning defects, such as OFT malformations, that have been also observed in human patients, giving a direct role to *Hoxa1* in OFT patterning (Makki and Capecchi, 2012). Additionally, mice lacking *Hoxb1* display OFT and ventricular septum (wall separating the two ventricles) defects. In this case, *Hoxb1* mutation led on the one hand to the upregulation of *fgf8* levels and abnormal SHF proliferation and on the other hand the upregulation of the SHF differentiation markers, α-actinin and MF20 and thus premature SHF differentiation. Furthermore, in compound *Hoxa1*, *Hoxb1* mutant mice, the OFT and ventral septum deficits were exacerbated, suggesting a genetic interaction between them (Roux et al., 2015). Interestingly, not only *Hoxb1* loss of function but also its overexpression leads to cardiac malformations (Zaffran et al., 2018). Extending this concept further, transcriptional profiling has shown that *Hoxb1* activates the posterior program of the SHF and inhibits the premature differentiation of progenitors by directly repressing *Natriuretic peptide precursor A* (*Nppa*) and *B* (*Nppb*) expression (Stefanovic et al., 2020).

Anterior Hox genes also play a role in cardiac NCC (Chisaka and Capecchi, 1991; Roux et al., 2017). *Hoxa3*-mutant mice display defects in carotid arteries as well as defaults in size and shape of heart compartments (Chisaka and Capecchi, 1991). *Hoxa1* and *Hoxb1* are required for cardiac NCC migration and their mutation leads to subsequent large artery patterning and outflow septum defects (Roux et al., 2017). Interestingly, an *in vitro* study using mouse embryonic stem cells has demonstrated a role for *Hoxa10* in the timing of cardiac cell differentiation, suggesting an unexpected role for posterior Hox in vertebrate cardiac development (Behrens et al., 2013). In contrast to *Drosophila* heart development, Hox PBC/MEIS cofactors were found to be associated with Hox function in vertebrates (Lescroart and Zaffran, 2018).

## Visceral Muscle

### Hox Control of *Drosophila* Visceral Musculature

The *Drosophila* gut is formed by the assembly of cells originating from all three germ layers; ectodermal, endodermal and mesodermal cells. The mesodermal visceral muscles are located in the external midgut layer surrounding the endoderm and are responsible for peristaltic movements. Five Hox genes (*Scr*, *Antp*, *Ubx*, *abd-A*, and *AbdB*) are expressed in the midgut visceral mesoderm and all except *Scr* and *AbdB* pattern the unsegmented gut together with ectodermal cues. Their expression in this tissue is parasegmental (*Scr* and *AbdB* excepted), and non-overlapping (a parasegment is a metameric unit composed of a posterior part of one segment and an anterior part of the adjacent segment). *Scr* mostly overlaps with PS4, *Antp* is expressed in PS5 and 6, *Ubx* in PS7, *abd-A* in PS8-12 and *AbdB* in the end of the midgut (Figure 1D). Curiously, there is a one-to-two cell gap between *Scr* and *Antp* expression domains. In the gut inner endoderm only *labial* (*lab*) expression can be detected (Diederich et al., 1989). Antp, Ubx, and AbdA are responsible for three midgut constrictions establishment, subdividing the midgut into four distinct chambers and seemingly helping to the proper gut elongation (Figure 1D). In homozygous *Antp* mutant embryos, the first constriction doesn’t form (Tremml and Bienz, 1989; Reuter and Scott, 1990). The establishment of the second constriction is dependent on both Ubx and AbdA. The third constriction is fully specified by AbdA (Tremml and Bienz, 1989). Scr is not involved in midgut subdivision, but in *Scr* mutant conditions, four protrusions located in the anterior midgut called gastric caeca do not form (Reuter and Scott, 1990). Finally, AbdB is required for the gut left-right asymmetry establishment by controlling the activity of a gene encoding for the type ID unconventional myosin (*myosinID*), a function presumed to be independent of Hox patterning function (Coutelis et al., 2013; Figure 1D).

Concerning Hox target genes in the visceral mesoderm, it was first predicted that Hox could regulate cytoskeleton or genes able to drive mesodermal cell contraction around the underlying endoderm, explaining the constriction establishment (Reuter and Scott, 1990). One such gene was identified, *beta3-tubulin*, encoding a cytoskeleton-associated protein whose expression is regulated by Ubx (Hinz et al., 1992). Importantly, Ubx is required for *decapentaplegic* (*dpp*) expression in the visceral mesoderm and together with AbdA controls *wingless* (*wg*) expression (Immerglück et al., 1990). The *cis*-regulatory region in the *dpp* gene regulated directly by Ubx and AbdA has been successfully identified and constituted the first example of a Hox target gene enhancer (Capovilla et al., 1994). Later, the enhancer in *wg* gene bound by AbdA and Mad (Mothers against dpp, transcription factor and Dpp signaling target), driving its expression was also identified (Grienenberger et al., 2003). In this particular case, the sole AbdA binding without Dpp input does not allow *wg* activation, once again demonstrating that a cooperative binding between Hox and transcription factors is required to convey a proper cell fate. All the following visceral mesoderm target genes identified are activated by Wg or Dpp and are thus indirect targets: In the anterior midgut mesoderm, Antp regulates *teashirt* (*tsh*) expression and Ubx, AbdA, Wg, and Dpp regulate its expression in the central part (Mathies et al., 1994). Through Wg and Dpp signaling, Ubx and AbdA activate *pointed* (*pnt*) and *odd-paired* (*opa*) in the specific posterior midgut mesodermal regions (Bilder et al., 1998).

### Hox Control of Vertebrate Visceral Musculature

In vertebrates as in *Drosophila*, the gut develops both from endoderm and mesoderm. More precisely, it develops from the splanchnic mesoderm, itself derived from the lateral plate mesoderm. The splanchnic mesoderm and the endoderm involute to form a primitive gut tube. The tube develops further into foregut, midgut comprising the small intestine, cecum and anterior portion of the large intestine and hindgut comprising the remainder of the large intestine and rectum. Hox genes are collinearly expressed along the lateral plate mesodermal component of the gut but also in the endoderm and the expression of many persists in the adult (Beck et al., 2000; Beck, 2002). The detailed, complex Hox expression patterns in different organs of the gut mesoderm has been extensively summarized previously (Beck et al., 2000; Choi et al., 2006).

Few Hox functions have been identified in the mouse gut, notably because of the co-expression in the same gut regions of two or more Hox from different paralogous groups, leading to high functional redundancy. Despite this, it has been shown that *Hoxd13* and *Hoxd12* are required for the generation of the anal sphincter (Kondo et al., 1996). When all *Hoxd* genes are deleted (except *Hoxd1* and *Hoxd3*) the ileocecal sphincter (separating small and large intestine) doesn’t form and the ileocecal smooth-musculature is disorganized (Zákány and Duboule, 1999). Hox genes are thus clearly required to pattern the unsubdivided gut mesoderm both in *Drosophila* and vertebrates. Similarly, deletion of the anterior part of the *HoxD* locus (from *Hoxd1* to *Hoxd10*) provokes agenesis of the caecum (at the junction of the ileum and large intestine). Interestingly, it has been demonstrated that it is not the combined deletion of these genes but instead the resulting strong ectopic *Hoxd12* expression that accounts for this phenotype (Zacchetti et al., 2007). Trying to find a common framework for the role of the different *HoxD* genes, it has been recently reported that *Hoxd3* deletion alone results in gut growth deficit, giving an essential role in the gut development to this Hox gene (Zakany et al., 2017).

A role has also been attributed to Hox genes from other paralogous groups, such as *Hoxc4*, whose deletion results in esophageal musculature disorganization and obstruction of the organ (Boulet and Capecchi, 1996). Mice carrying a *Hoxa4* transgene, resulting in its strong overexpression in the gut, develop a congenital megacolon phenotype characterized by a largely distended colon (Wolgemuth et al., 1989). Deletion of *Hoxa5* leads to stomach morphogenesis defaults, presumably by controlling the epithelio-mesenchymal signaling. Indeed, in the *Hoxa5*-deficient mice stomach epithelium, the expression pattern of *Ihh*, *Shh*, and *Fgf10* changed and the expression levels of *Ptc* and *Gli* increased (Aubin et al., 2002). Misexpression of *Hoxc8* under the control of *Hoxa4* regulatory elements, resulting in a shift in the anterior boundary of its expression, results in several hamartomatous lesions, where gastric epithelium was found embedded within the stomach musculature (Pollock et al., 1992).

## Concluding Remarks

Hox genes are involved in the patterning of all muscle types in *Drosophila* and vertebrates. While how they achieve this is not completely resolved, plethora studies in different model organisms have gone a long way to determine their role in the numerous muscle types. One emerging concept would appear that Hox act at numerous stages of muscle development, where at early stages, Hox appear to play a specifying role by providing spatial cues along the anterior–posterior axis, and at later stages controlling basic cellular functions such as proliferation, cell survival, death etc. This follows the role Hox play in patterning of the ectoderm and so it is perhaps without surprise that Hox contribute similarly in muscle development through mesoderm patterning.

As in patterning of the ectoderm, in muscle development there is often requirement for the cooperation between Hox and other tissue-specific transcription factors, which are themselves Hox target genes together with signaling molecules. Examples about Hox functional conservation can be found mainly in vertebrate cardiac muscle, but conservation also exists in skeletal and gut muscles, further suggesting a universal role for Hox in mesodermal patterning. While how this achieved is not fully understood, knowledge gained in how Hox generate diversity in the CNS should help understand their role in generating different muscle types, which together allow for the development of more complex organisms.

Even though a large number of Hox-dependent functions across different mesodermal derivatives are now known, there is however only sparse evidence about the underlying molecular mechanisms. To fully understand how Hox orchestrate muscle development, it is essential to define the network of genes they regulate, in addition to the tissue-specific transcription factors such as the identity TFs in muscle and temporal TFs in the CNS. The discovery of novel *cis*-regulatory regions of Hox target genes was historically a difficult and laborious process, but now with the vast advances in genome-wide approaches, both spatially and temporally, at the level of whole tissue or single cell, it is soon possible to better define Hox regulatory regions and target genes. Such a genome-wide spatio-temporal approach will thus allow us to fully grasp the complex and intricate networks defining how Hox proteins regulate muscle development.

## Author Contributions

GP designed and drew illustrations, and wrote the manuscript with inputs from CM-Z, YG, and AS. All authors contributed to the article and approved the submitted version.

## Conflict of Interest

The authors declare that the research was conducted in the absence of any commercial or financial relationships that could be construed as a potential conflict of interest.

## Publisher’s Note

All claims expressed in this article are solely those of the authors and do not necessarily represent those of their affiliated organizations, or those of the publisher, the editors and the reviewers. Any product that may be evaluated in this article, or claim that may be made by its manufacturer, is not guaranteed or endorsed by the publisher.
